# Ultrasound-Assisted Intraosseous Injection of Platelet-Rich Plasma for a Patient With Tibial Plateau Subchondral Bone Marrow Lesion: A Case Presentation and Technical Illustration

**DOI:** 10.7759/cureus.12312

**Published:** 2020-12-26

**Authors:** King Hei Stanley Lam, Chen-Yu Hung, Andy Hung

**Affiliations:** 1 Department of Family Medicine, The Chinese University of Hong Kong, Hong Kong, CHN; 2 Physical Medicine and Rehabilitation, National Taiwan University Hospital Beihu Branch, Taipei, TWN; 3 Regenerative Medicine, Shen-Shen Regenerative Medicine Clinic, Taipei, TWN

**Keywords:** ultrasound-guided, knee osteoarthritis/ koa, bone marrow injection, platelet-rich plasma/ prp, intervention pain physician

## Abstract

The subchondral bone marrow lesion (BML) has been found to have a significant correlation with pain in osteoarthritis patients. The intraosseous injection with orthobiologics such as platelet-rich plasma (PRP) or bone marrow aspirate concentrate has shown a promising therapeutic effect on BML-related pain. Traditionally, the intraosseous injection was performed with a large caliber trocar to break into the subchondral bone under fluoroscopy guidance and the patient was usually sedated prior to the procedure. In this report, we presented a 55-year-old woman who suffered from refractory right lateral knee pain for three months. The MRI revealed a right lateral tibial plateau subchondral BML, tears of medial and lateral menisci, and a general osteoarthritic appearance. We used ultrasound (US) to guide a 21-gauge needle through a pre-existing cortical break on the Gerdy’s tubercle for the intraosseous PRP injection. The contrast was confirmed to reach the subchondral bone of the lateral tibial plateau and the injection reproduced the patient's symptoms. Three weeks later, the patient had significant improvement in the visual analog scale and function. In conclusion, intraosseous injection with PRP is a possibly effective treatment for subchondral BML in knee osteoarthritis, and US can facilitate a smaller gauge needle placement without the need to sedate the patient.

## Introduction

Osteoarthritis is commonly regarded as a degenerative process that involves the damage and loss of the articular cartilage and progressive destruction of the intraarticular structures, leading to pain, impaired joint function, and decreased quality of life [1]. Articular cartilage lesions are usually accompanied by subchondral bone alteration or bone marrow lesions (BMLs) which are diagnosed using magnetic resonance imaging (MRI) with high signal intensity on fluid-sensitive sequences with or without low T1-weighted signals [1]. The presence of BML has been strongly associated with osteoarthritis pathogenesis and pain in knee osteoarthritis [2]. Therefore, the subchondral BMLs have been considered as one of the treatment targets in knee osteoarthritis patients and recent studies have shown a positive therapeutic effect with intraosseous platelet-rich plasma (PRP) or bone marrow aspirate concentrate injections [1,3]. The traditional intraosseous injection is performed by using a large caliber trocar under fluoroscopy. However, radiation exposure can be a limitation for this procedure [2]. Also, using a trocar to break into the subchondral bone can be destructive for the overlying cortical bone and the patient is usually sedated prior to the procedure. In recent years, ultrasound (US) has emerged as the first-line imaging modality for musculoskeletal conditions for its accessibility, portability, lower cost, lack of radiation, and allowance of real-time assessment and guided interventions [4]. In this regard, we would like to report a case with lateral knee pain caused by lateral tibial plateau BML and the utility of US to assist a small gauge needle placement through a pre-existing cortical break without the need to sedate the patient.

## Case presentation

**Figure 1:**
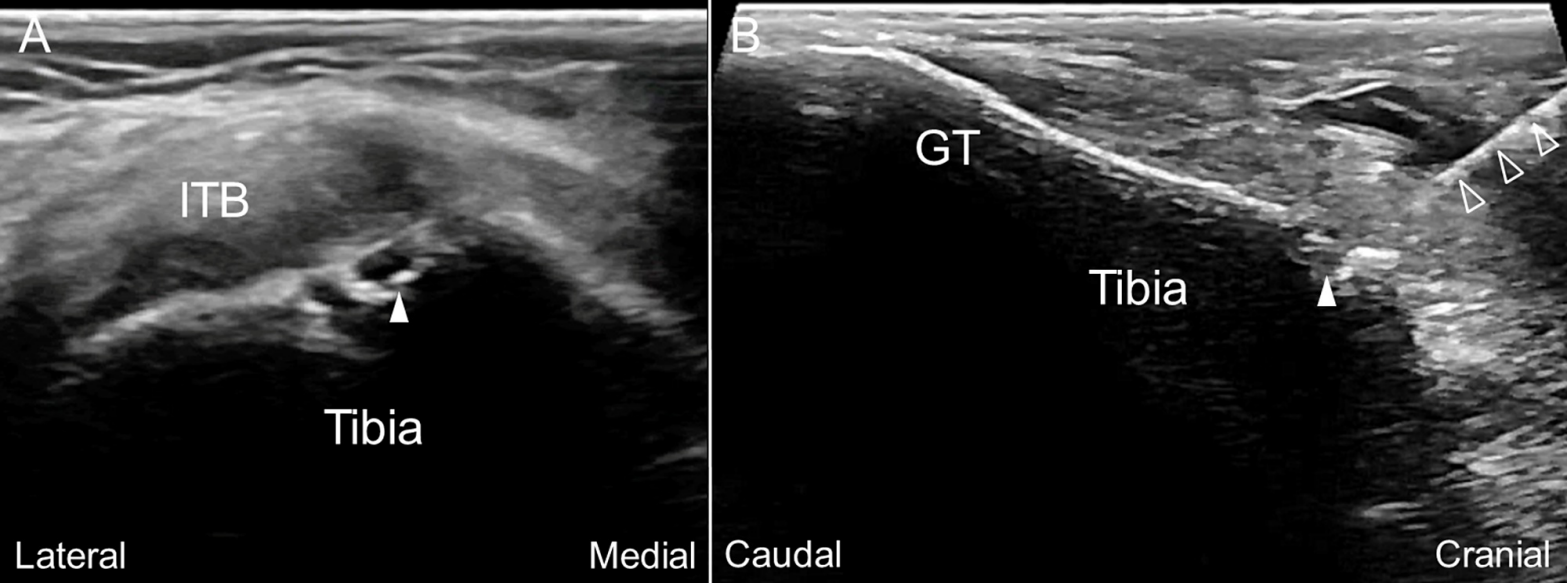
Ultrasound imaging over the lateral tibial plateau of the patient's symptomatic knee joint The short (A) and long (B) axis ultrasound (US) image over the lateral tibial plateau near the Gerdy’s tubercle (GT) revealed a cortical break (arrowhead) under the iliotibial band (ITB). US-guided in-plane injection aiming the cortical break was performed with the needle (void arrowheads) trajectory from cranial to caudal (B).

**Figure 2:**
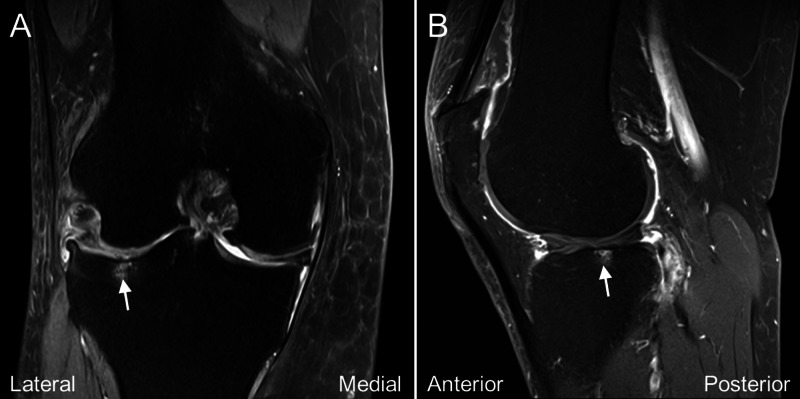
Magnetic resonance imaging of the patient's symptomatic knee joint before treatment The coronal (A) and sagittal (B) fat-sat T2 weighted magnetic resonance imaging of the knee joint revealed subchondral bone marrow high signal intensity (arrow) of the lateral tibial plateau along with medial and lateral meniscal tears.

A 55-year-old woman complained of right knee pain off and on for years. The pain was located mainly over the lateral knee and was noted when she bore weight on her right lower limb, such as standing up from bed in the morning, standing for more than 30 minutes for housework, walking, and stair climbing. There was occasional right knee swelling if she walked for a long distance. No resting pain or nocturnal pain was complained. Osteoarthritis of the right knee joint (Kellgren and Lawrence classification grade III) was diagnosed at another clinic. The pain worsened three months before her visit, and she failed to improve with the conservative treatment, including three courses of physical therapy with physical modality and exercise, and acupuncture three times weekly for two months. The visual analog scale (VAS) was 7-8/10 and the Western Ontario and McMaster Universities Arthritis Index (WOMAC) scores were 16/20 for pain, 4/8 for stiffness, 51/68 for physical disability. On physical examination, she had an antalgic gait and a slight reduction of range of motion of the right knee compared to the left (right 5-110 degrees and left 0-120 degrees). There was severe tenderness (VAS 7-8/10) over the lateral part of the tibial plateau, which in fact reproduced the patient’s usual pain when she stepped down on the floor each morning. McMurray test was positive over both the medial and lateral menisci. The US examination revealed signs of lateral and medial meniscus tears with marginal osteophytes and a cortical break over the lateral tibial plateau close to the Gerdy’s tubercle (Figure 1). The MRI revealed a general osteoarthritic change and tears of the lateral and medial menisci, and there was a subchondral BML over the lateral tibial plateau (Figure 2). After a discussion about the treatment, the patient opted for regenerative injection therapy.

**Figure 3:**
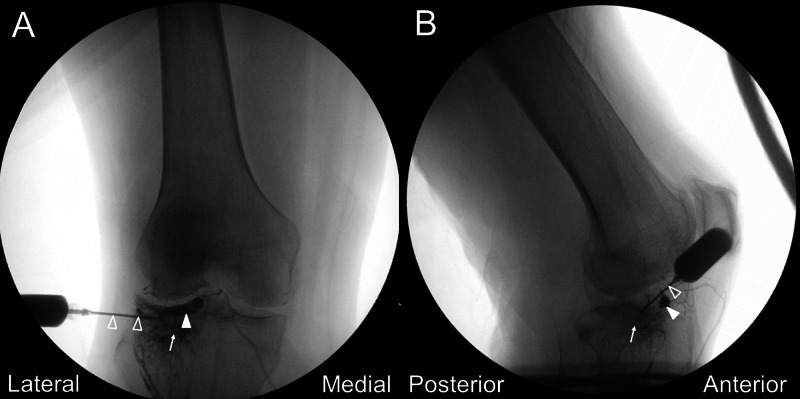
Fluoroscopic views following contrast injection The anteroposterior (A) and lateral (B) fluoroscopic views confirmed the contrast to be within the lateral tibial plateau (arrow). Arrowhead, contrast within the supporting artery along the lateral meniscus; void arrowhead, needle shaft.

**Figure 4:**
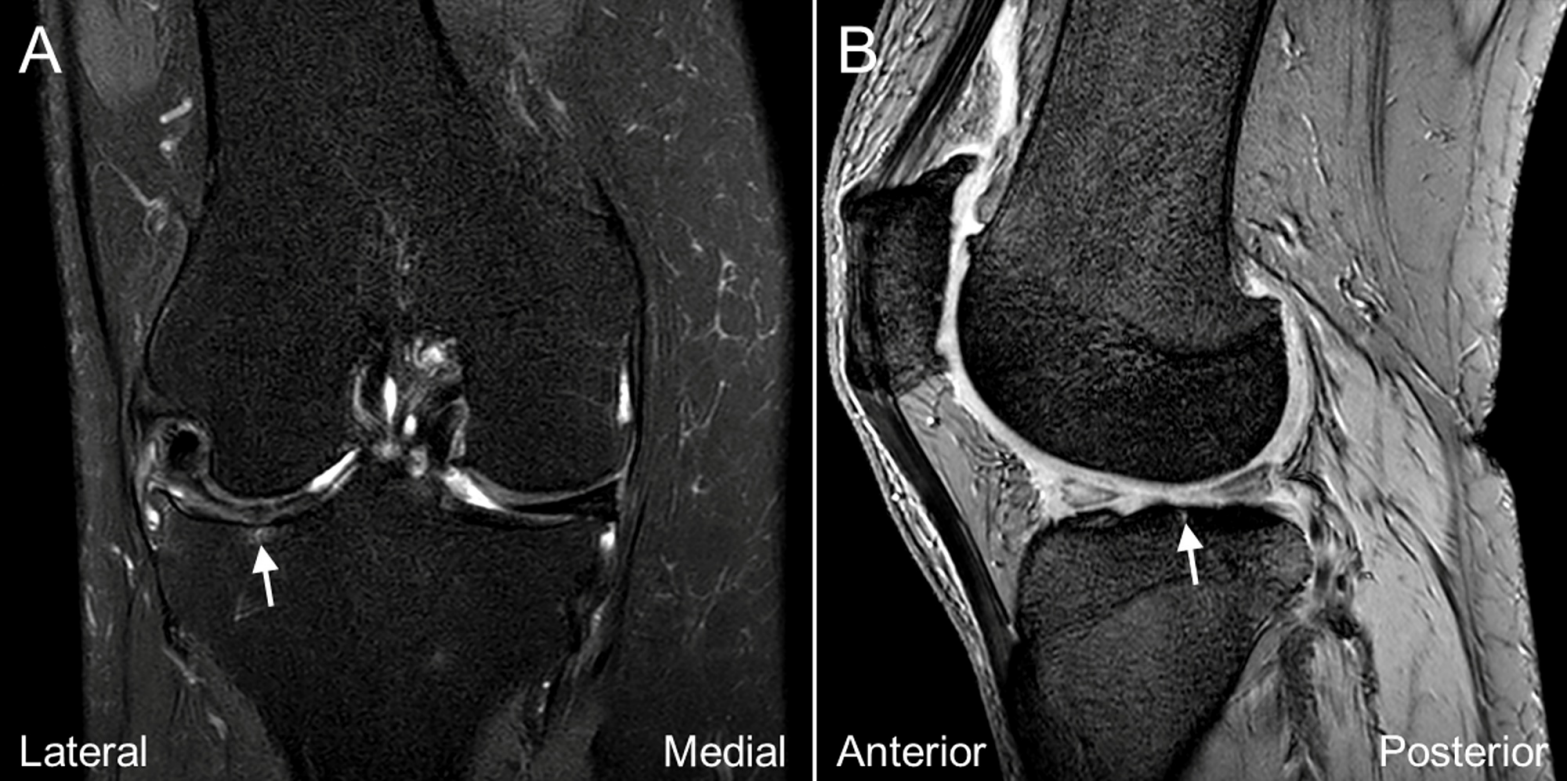
Magnetic resonance imaging of the patient's symptomatic knee joint six months after treatment The coronal (A) and sagittal (B) fat-sat T2 weighted magnetic resonance imaging of the knee joint revealed resolution of the subchondral bone marrow lesion (arrow).

We performed a US-assisted intraosseous injection of PRP (Smart Prep II; Harvest Technologies, Belton, TX, USA; 60ml whole blood to 6ml PRP) at the subchondral BML of the lateral tibial plateau by placing a 21-gauge, 1.5-inch needle through the cortical break close to the Gerdy’s tubercle (Figure 1B, Video 1). After evaluating the orientation of the opening of the cortical break, the needle was inserted into the apex of the hole and further advanced deeper to the subchondral bone. The contrast (Ominpaque 350) was then injected under fluoroscopy to confirm the needle placement and evaluate the contrast spread (Figure 3). The contrast reached the location of the subchondral bone marrow lesion at the lateral tibial plateau and the injection did reproduce the patient’s usual knee pain on weight-bearing. PRP injection (3ml) was then offered to the same region and the displacement of the contrast was observed under fluoroscopy. The patient was kept to have non-weight-bearing to her right knee for two days and when she was allowed to have full-weight bearing walking on the third day, she reported VAS 3/10. Three weeks later, the patient reported significant improvement of her pain with VAS 0-2/10. She could walk 1.5 to two hours over level ground and stand more than 1.5 hours without pain. The WOMAC scores were 4/20 for pain, 0/8 for stiffness, 12/68 for physical disability. The MRI six months after the treatment revealed resolution of the subchondral BML (Figure 4).

**Video 1:** Intraosseous Injection of Knee Bone Marrow Lesion through a Cortical Break on Lateral Tibial Plateau

## Discussion

The subchondral BMLs are found to be associated with a wide range of pathological conditions, such as trauma, osteoarthritis, insufficiency fracture, and osteonecrosis [1,5]. Histologically, these lesions may correspond to bone marrow edema, microfracture, fibrosis, or cyst formations [5]. Studies have found these changes to be associated with subchondral bone attrition and loss of mechanical integrity, leading to the progression of cartilage loss and rapid joint deterioration [3]. Therefore, the subchondral BMLs represent a new possible target to be addressed for the treatment of osteoarthritis. A recent systematic review on knee intraosseous injection found the procedure to be safe and beneficial to address subchondral bone damage in the short-term follow-up. However, the current evidence is not able to identify the superiority of a specific product for injection over the others [6]. Traditionally, the procedure utilized fluoroscopy to determine the injection site and the distribution of injectate. A large caliber trocar was used to break through the bony cortex and conscious sedation is generally administered prior to the procedure [2]. In our method, we used US to guide a smaller needle (usually 21- to 25-gauge, 1.5 to 2 inches long) through a pre-existing cortical break proximal to the Gerdy's tubercle for the intraosseous injection to the BML at the lateral tibial plateau. In the authors’ experience, these cortical breaks are usually observed near the subchondral bone where the ligament/capsule/tendon attach. We speculated that they might result from the preceding mechanical traction injury by these soft tissue structures. Also, the cortical break could be the opening of the subchondral BML when the lesion was severe and extended to the surface of the bony cortex. By using a pre-existing bony opening as the portal, this technique prevented making another bony cortex defect. Moreover, when the needle was introduced to the cortical break and advancing further into the bone, the physician could use the needle to mechanically stimulate the cleft to see if the patient’s usual pain was reproduced. Since a smaller needle was used, the pressure effect during the injection was less, and the patient could usually tolerate the intraosseous injection pain without the need for sedation as in the traditional procedure.

For a successful US-assisted intraosseous needle placement, several technical details have to be emphasized. First, the power Doppler mode should be performed before the needle entrance. This is essential as a hole of the bony cortex with positive power Doppler flows is probably a nutrient foramen with nutrient vessels or where the epiphyseal/metaphyseal vessels enter the bone [7]. In these cases, the bony opening is not suitable to access for intraosseous injection. Second, the physician should evaluate the cortical break with biplanar images to determine the orientation of the hole and plan the trajectory. Third, unlike other US-guided interventions, an out-of-plane injection technique is exceptionally favored in the US-assisted intraosseous injection as the needle trajectory is more perpendicular to the bony cortex and easier to get to the apex of the hole and enter the subchondral bone [8,9]. In our patient, however, we adopted an in-plane approach as the opening of the cortical break proximal to the Gerdy's tubercle is angled and not perpendicular to the skin.

## Conclusions

The subchondral BMLs could be the pain generator in patients suffering from knee joint pain. The intraosseous injection with PRP is a possibly effective treatment for subchondral BML, and the US can facilitate needle placement using a smaller gauge needle without the need to sedate patients.

## References

[1] Lychagin A, Lipina M, Garkavi A (2020). Intraosseous injections of platelet rich plasma for knee bone marrow lesions treatment: one year follow-up. Int Orthop.

[2] Delgado D, Garate A, Vincent H (2019). Current concepts in intraosseous platelet-rich plasma injections for knee osteoarthritis. J Clin Orthop Trauma.

[3] Kasik CS, Martinkovich S, Mosier B, Akhavan S (2019). Short-term outcomes for the biologic treatment of bone marrow edema of the knee using bone marrow aspirate concentrate and injectable demineralized bone matrix. Arthrosc Sports Med Rebil.

[4] Chiu YH, Chang KV, Chen IJ, Wu W-T, Özçakar L (2020). Utility of sonoelastography for the evaluation of rotator cuff tendon and pertinent disorders: a systematic review and meta-analysis. Eur Radiol.

[5] Kon E, Ronga M, Filardo G (2016). Bone marrow lesions and subchondral bone pathology of the knee. Knee Surg Sports Tramatol Arthosc.

[6] Di Matteo B, Polignano A, Onorato F (2020). Knee intraosseous injections: a systematic review of clinical evidence of different treatment alternatives. Cartilage.

[7] Longia GS, Ajmani ML, Saxena SK, Thomas RJ (1980). Study of diaphyseal nutrient foramina in human long bones. Acta Anat.

[8] Smith J, Finnoff JT (2009). Diagnostic and interventional musculoskeletal ultrasound: part 1. Fundamentals. PM R.

[9] Tirado A, Nagdev A, Henningsen C, Breckon P, Chiles K (2013). Ultrasound-guided procedures in the emergency department: needle guidance and localization. Emerg Med Clin North Am.

